# Omics analyses and biochemical study of *Phlebiopsis gigantea* elucidate its degradation strategy of wood extractives

**DOI:** 10.1038/s41598-021-91756-5

**Published:** 2021-06-15

**Authors:** Mana Iwata, Ana Gutiérrez, Gisela Marques, Grzegorz Sabat, Philip J. Kersten, Daniel Cullen, Jennifer M. Bhatnagar, Jagjit Yadav, Anna Lipzen, Yuko Yoshinaga, Aditi Sharma, Catherine Adam, Christopher Daum, Vivian Ng, Igor V. Grigoriev, Chiaki Hori

**Affiliations:** 1 Graduate School of Chemical Sciences and Engineering, Hokkaido University, Sapporo, 080-682 Japan; 2 CSIC, Instituto de Recursos Naturales y Agrobiología de Sevilla (IRNAS), Reina Mercedes 10, 41012 Seville, Spain; 3 University of Wisconsin Genetics Biotechnology Center, Madison, WI 53706 USA; 4 Forest Products Laboratory, USDA, Madison, WI 53726 USA; 5 Department of Biology, Boston University, Boston, MA 02215 USA; 6 University of Cincinnati, Cincinnati, OH 45267 USA; 7 Lawrence Berkeley National Laboratory, US Department of Energy Joint Genome Institute, Berkeley, CA 94720 USA; 8 Department of Plant and Microbial Biology, University of California Berkeley, Berkeley, CA 94720 USA; 9 Division of Applied Chemistry, Department of Engineering, Hokkaido University, Sapporo, Hokkaido 060-8628 Japan

**Keywords:** Microbiology, Fungi

## Abstract

Wood extractives, solvent-soluble fractions of woody biomass, are considered to be a factor impeding or excluding fungal colonization on the freshly harvested conifers. Among wood decay fungi, the basidiomycete *Phlebiopsis gigantea* has evolved a unique enzyme system to efficiently transform or degrade conifer extractives but little is known about the mechanism(s). In this study, to clarify the mechanism(s) of softwood degradation, we examined the transcriptome, proteome, and metabolome of *P. gigantea* when grown on defined media containing microcrystalline cellulose and pine sapwood extractives. Beyond the conventional enzymes often associated with cellulose, hemicellulose and lignin degradation, an array of enzymes implicated in the metabolism of softwood lipophilic extractives such as fatty and resin acids, steroids and glycerides was significantly up-regulated. Among these, a highly expressed and inducible lipase is likely responsible for lipophilic extractive degradation, based on its extracellular location and our characterization of the recombinant enzyme. Our results provide insight into physiological roles of extractives in the interaction between wood and fungi.

## Introduction

The most abundant source of terrestrial carbon is woody plant biomass. The cell wall largely consists of cellulose, hemicellulose and lignin, the proportions of which differ between hardwood and softwood species^1,2^. In addition to these structural components, a solvent-soluble fraction, commonly referred to as extractives, constitute up to 10 w/w% of the total plant biomass, though the chemical compounds vary with tree species, age and position, i.e. bark, heartwood or sapwood (inner xylem or outer xylem, respectively)^3–5^. Extractives are considered to be a factor impeding or excluding fungal colonization of the freshly harvested conifers^6^. The ligninolytic basidiomycete *Phlebiopsis gigantea* can rapidly invade such softwood^7^ but little is known about the mechanism(s) of how *P. gigantea* could contend with the extractives from the plant^8,9^. A measure of this efficient softwood colonization has been the use of *P. gigantea* inoculum to reduce the spread of *Heterobasidion* root rot on conifer stumps^10,11^. Thus, *P. gigantea* has evolved a unique system to efficiently transform and/or utilize conifer extractives.

Consistent with the observed efficient utilization of lignocellulosic materials, genome analyses shows that *P. gigantea* possesses hundreds of genes encoding carbohydrate active enzymes (CAZy) for plant cell wall depolymerization and utilization^12^. Our recent proteomic analyses demonstrated that *P. gigantea* employs a suite of classical cellulases, hemicellulases, and lignin-degrading enzymes when colonizing freshly cut pine in the presence of various extractives^13^. The transcriptome profiles further suggest that *P. gigantea* processes coniferous extracts by using ATP-binding cassette (ABC) transporter and a glutathione S-transferase similar to other fungi. For example, a set of genes encoding an efflux ABC transporter in *Grosmannia clavigera* is thought to confer monoterpene-tolerance^14^, and a glutathione S-transferase in *Phanerochaete chrysosporium* provides antioxidant activity^15^. Other genes are suggested to function in lipid metabolism by *P. gigantea* but our knowledge of specific genes involved in the metabolism(s) of coniferous extractives by this fungus remains uncertain in large part due to the complexity of the woody substrate^13^. In short, it is not clear whether the coniferous extractives specifically induced the observed metabolic pathways in *P. gigantea*.

Free and esterified fatty acids together with resin acids are major components of softwood extractives, and basidiomycetes are known to secrete lipases that hydrolyze glycerides to release fatty acids^13^. However, how this group of fungi regulates the genes involved in utilizing lipids in the wood extractives remains unknown. Here, we attempt to elucidate the extractive-induced mechanism(s) of *P. gigantea*. We cultivated the fungus on a defined culture medium containing microcrystalline cellulose as a carbon source and supplemented with different amounts of the *Pinus taeda* sapwood extracts, which contained high levels of the lipids. Metabolites of lipophilic extractives in the culture supernatant were determined by GC–MS. Transcriptomic analyses of the grown mycelia and proteomic analyses of the supernatants were performed by RNA-seq and LC–MS/MS, respectively, to identify genes encoding enzymes related to transformation or degradation of the extractives by *P. gigantea*. A putative extracellular lipase was highly expressed and regulated in response to the extractives. This enzyme was produced in the heterologous expression system of *Pichia pastoris*, and functionally characterized as a novel basidiomycete lipase.

## Results

### Chemical composition of *P. taeda *wood extractives during the growth of *P. gigantea*

**Figure 1:**
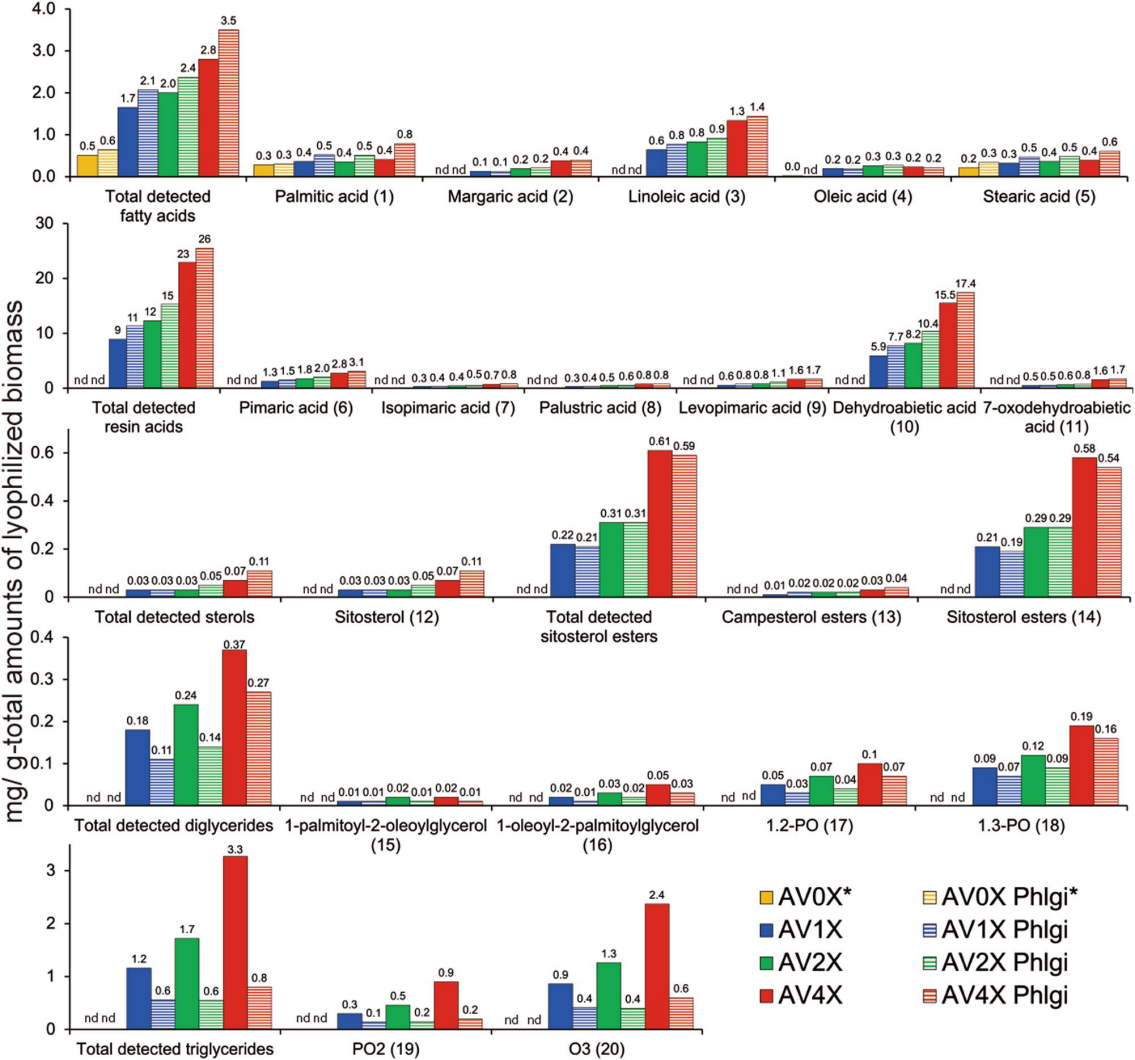
Chemical composition of lipophilic compounds in lyophilized Avicel microcrystalline cellulose medium containing none (AV0X) or increasing amounts of loblolly pine extract (AV1X, AV2X, AV4X) without and with *P. gigantea* inoculation by GC–MS analyses (n = 1). The culture media were harvested five days after the inoculation (AV0X Phlgi, AV1X Phlgi, AV2X Phlgi, AV4X Phlgi) (n = 1). Numbers in parentheses correspond to peaks shown in GC–MS chromatograms (Figure S1). *The fatty acids present in control samples (AV0X and AV0X Phlgi) were thought to be contaminants arisen during sample preparation.

The chemical composition of the 70 v/v% acetone-treated extractives from the grounded powder of *P. taeda* sapwood was analyzed by GC–MS (Fig. 1; Fig. S1). In this GC–MS analysis, a constant amount of microcrystalline cellulose (Avicel) was supplemented with the following series of *P. taeda* extractives, in the absence of extractives (AV0X), the equivalent amount of extractives present in the *P. taeda* wood (AV1X), twofold (AV2X), and fourfold *P. taeda* extractives (AV4X), which were also used as the culture medium of *P. gigantea*. The 20 representative compounds (Fig. 1; AV0X, AV1X, AV2X, and AV4X) were determined. The fatty acids present in control sample (AV0X) were thought to be contaminants arisen during sample preparation. The respective concentration of sample components generally increased with more concentrated extracts except for some fatty acids, which was probably due to contaminants. As expected, this included most of the detectable fatty and resin acids, diglycerides, free sterols and sterol esters, and triglycerides^16^. Culture media with the same composition were also analyzed after 5 days growth (Fig. 1; AV0X Phlgi, AV1X Phlgi, AV2X Phlgi, and AV4X Phlgi). When the medium was inoculated with *P. gigantea*, it grew on media containing microcrystalline cellulose, and the levels of di- and triglycerides appeared to decrease (Fig. 1) whereas fatty and resin acids, and sterols were unaffected after the 5-days cultivation. For some compounds, amounts per total amounts of lyophilized biomass seems to increase likely due to the efficient consumption of microcrystalline cellulose by *P. gigantea.* Decrease of di- and triglycerides during growth of *P. gigantea* was hypothesized to be a response to the extractives, especially lipid compounds*.*

### Genome-wide gene expression profiles of *P. gigantea* in response to the extractives

**Figure 2:**
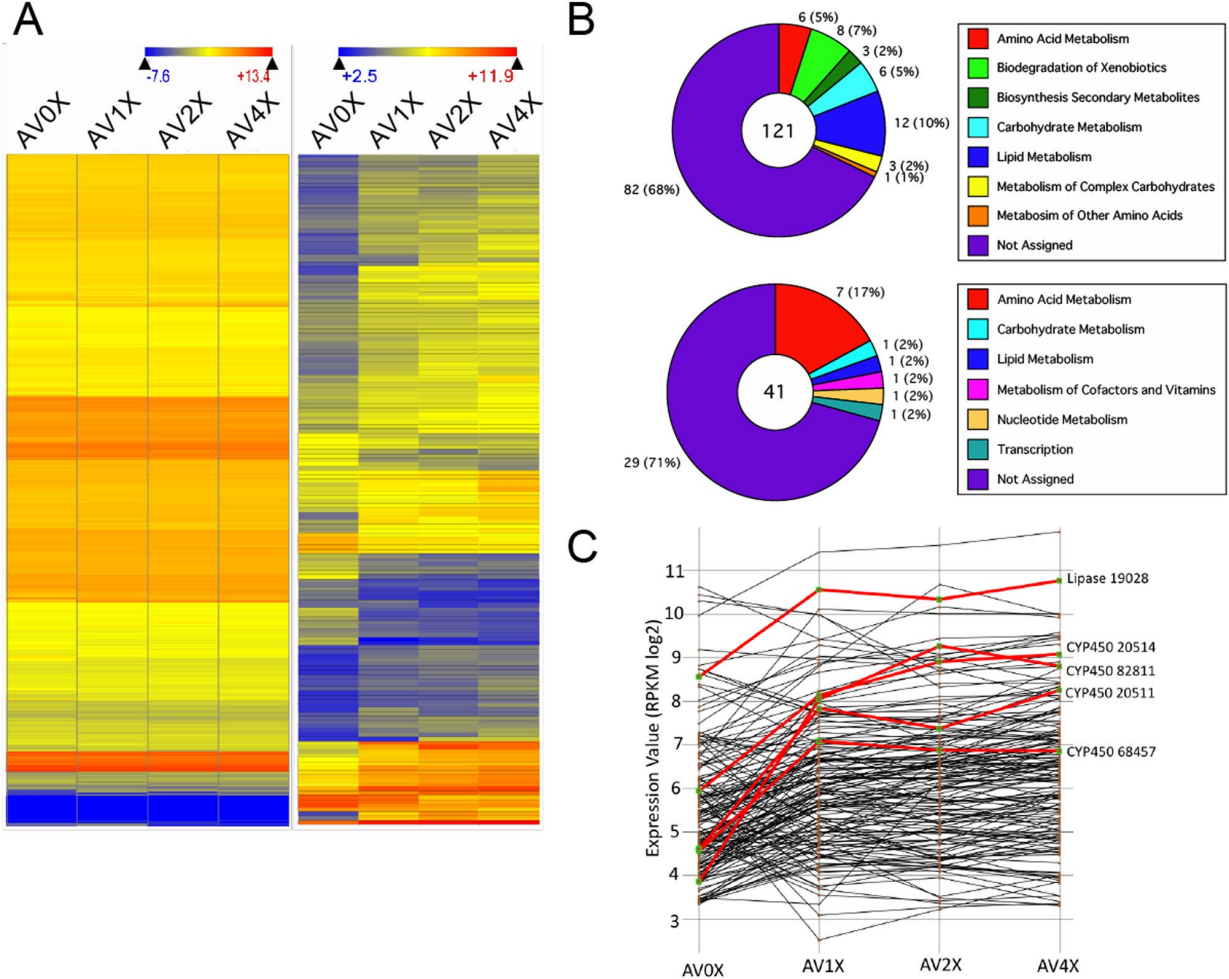
(**A**) Heat maps showing expression values of all 11,891 *P. gigantea* genes (left panel) and a subset of 161 significantly (*P* < 0.02) regulated genes (right panel) when cultured in microcrystalline cellulose medium containing none (AV0X) compared to increasing amounts of loblolly pine extract (AV1X, AV2X, AV4X). DNAStar’s hierarchical clustering program with euclidean distance and centroid linkage methods were used. (Gene tree not shown). (**B**) Functional classification of 161 *P. gigantea* genes whose transcripts levels increased (upper panel) or decreased (lower panel) relative to AV0X. Only those exhibiting > twofold change, *P* < 0.02 and RPKM values > 10 are shown. See Supplemental data file S2 for complete listing of Gene Ontology (GO) and related terms. (**C**) Line graph plot of signals for each of the 161 regulated transcripts observed in the four media. Highlighted transcripts corresponding to four cytochrome P450s and lipase protein model #19028 exhibiting significant accumulation (> fourfold) even at relatively low extract addition (AV1X).

To identify the extract-responsive genes of *P. gigantea*, the transcriptomes in the cells grown on the four culture media were analyzed by RNA-seq. Additionally, for comparison we collected a new RNA-seq dataset from cells grown on *P. taeda* wood powder (LPAS) including extractives. All transcript data, along with manual annotations, gene ontology terms (GO), and genome locations, are summarized in Supplemental data file S1. Of 11,891 genes in the genome of *P gigantea*, 11,437, 11,434, 11,431, 11,423 and 11,394 transcripts were identified in the cells grown on AV0X, AV1X, AV2X, AV4X and LPAS cultures, respectively. The average expression values (RPKMs)/standard deviation were 75.5/257, 73.8/236, 74.1/243, 77.6/243 and 80.5/345 for the datasets of AV0X, AV1X, AV2X, AV4X, and LPAS, respectively. With notable exceptions, transcript profiles of *P gigantea* genes were generally conserved in microcrystalline cellulose-based media AV0X, AV1X, AV2X, and AV4X (Fig. 2A, *left*) but diverged substantially from loblolly pine medium (LPAS) as described in Fig. S2. Microcrystalline cellulose induced various CAZymes involved in the complete deconstruction of lignocellulose and include highly expressed conventional cellulases and hemicellulases (e.g. belonging to families GH5-5, GH5-7, GH7, GH6, GH10, GH11) and lytic polysaccharide monooxygenases (LPMOs) (Table S1), and the expression of these CAZy genes was conserved between the microcrystalline cellulose containing media. In the presence of differing amounts of extractives (AV1X, AV2X, and AV4X), 121 and 41 transcripts were significantly up- and down-regulated relative to AV0X (*P* < 0.02, > twofold, RPKM ≥ 10) (Fig. 2A, *right*; Fig. S3; Supplemental data file S2). This total of 162 differently regulated genes can be expanded to 552 genes (406 up- and 146 down-regulated relative to the AV0X dataset), when the stringency is relaxed (*P* < 0.05, > twofold, RPKM ≥ 10; Fig. S3).

Among the genes encoding lignocellulose-degrading enzymes, only seven genes were up-regulated, and two were down-regulated when the extractives were present in the media (AV1X, AV2X, and AV4X), compared to that of no extractives (AV0X), respectively (*P* < 0.02, > twofold, RPKM ≥ 10) (Table 1). Under the less stringent threshold (*P* < 0.05, > twofold, RPKM ≥ 10), 16 and 10 transcripts were up-, and down-regulated, respectively, relative to the AV0X (Table S2). Dye peroxidase (Phlgi_78526) and AA5_1 copper radical oxidase (Phlgi_128606) were significantly down-regulated, while AA3_2 GMC oxidase (Phlgi_101126), GH18 chitinases (Phlgi_88507 and Phlgi_27927), GH11 xylanase (Phlgi_21241), GH12 glucanase (Phlgi_126344), and GH28 pectinase (Phlgi_28251 and Phlgi_36341) were significantly up-regulated. These results indicated that the CAZymes, which play central roles in lignocellulose degradation, such as GH7 and GH6 cellobiohydrolases/endo-glucanases, and AA2 manganese peroxidases (MnP) are not regulated by the presence of extractives. In contrast, a few genes encoding glycoside hydrolase (GH) families 11 endo-xylanase, GH12 endo-glucanase, and GH28 polygalacturonase were significantly up-regulated, suggesting that pectin and other polysaccharide hydrolysis is likely a first step of wood decay in response to extractives coated on wood material.

**Table 1:**
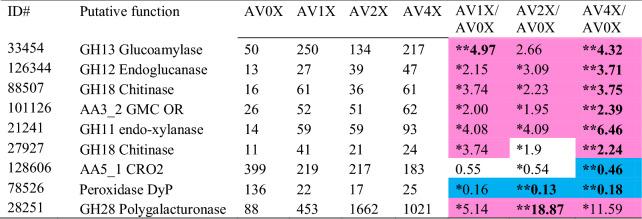
RPKM values and ratios of *P. gigantea* regulated genes involved in lignocellulose degradation.

**Bold: *P* value < 0.02, *P value < 0.05, Magenta > 2, Blue < 0.5.

The functional distribution of the highly up-regulated genes in the presence of the increasing amounts of extractives were involved in amino acid, lipid, xenobiotic, and carbohydrate metabolism based on GO classifications (Fig. 2B; Supplemental data file S2). The 121 significantly up-regulated genes included 12 genes in lipid metabolism, 8 genes in biodegradation of xenobiotics, 6 genes in carbohydrate metabolism, and several genes in tryptophan metabolism and the biosynthesis of secondary metabolites, including phenylpropanoids and alkaloids. Twelve genes classified into lipid metabolism such as β-oxidation-related proteins, included Phlgi_130767, Phlgi_27604, and Phlgi_27649 β-ketothiolases (KTs), Phlgi_126556 long fatty acid CoA ligase, Phlgi_18116, and Phlgi_91676 acyl-CoA dehydrogenases (ADH), and Phlgi_29221 and Phlgi_27759 enoyl-CoA hydratases (EHs). Among the 8 genes involved in biodegradation of xenobiotics such as cytochrome P450s, interestingly, four substantially up-regulated genes were annotated as cytochrome P450 monooxygenases, Phlgi_20511, Phlgi_20514, Phlgi_82811, and Phlgi_68457, (> fourfold, *P* < 0.02, RPKM values ≥ 10) in response to supplementation with pine extractive (Fig. 2C; Supplemental data file S2). The induced genes belonged to P450 families, including families CYP52 (CYP5150A), CYP53 (CYP53C), CYP67 (CYP5035A), and CYP503 (CYP512B), respectively^13^. Among the 41 significantly down-regulated genes, many were categorized as amino acid metabolism, but most were not assigned by GO terms.

Tolerance to terpenes may be mediated in part by a putative ABC efflux transporter (Phlgi1_130987, Supplemental data file S2). Of the 51 ABC transporters of *P. gigantea*, this protein is most closely related to the *GcABC-G1* gene of the ascomycete *Grosmannia clavigera*, a pathogen of *Pinus contorta*^14^. The *GcABC-G1* gene is upregulated in response to various terpenes and appears to be a key element against the host defenses. Consistent with a similar function, our analysis showed the *P. gigantea* homolog to be upregulated 11- to 20-fold relative to microcrystalline cellulose without extractives (Supplemental data file S2). Differential regulation also implicated glutathione S-transferase (Phlgi_101998) in the transformation and detoxification of extractives (Supplemental data file S2). Homologs of upregulated genes encoding aldehyde dehydrogenase (Phlgi_121047), and aryl-alcohol dehydrogenase (Phigi_89048) are induced by aromatic compounds in *P. chrysosporium*^17,18^.

### Lipid metabolism in *P. gigantea*

**Figure 3:**
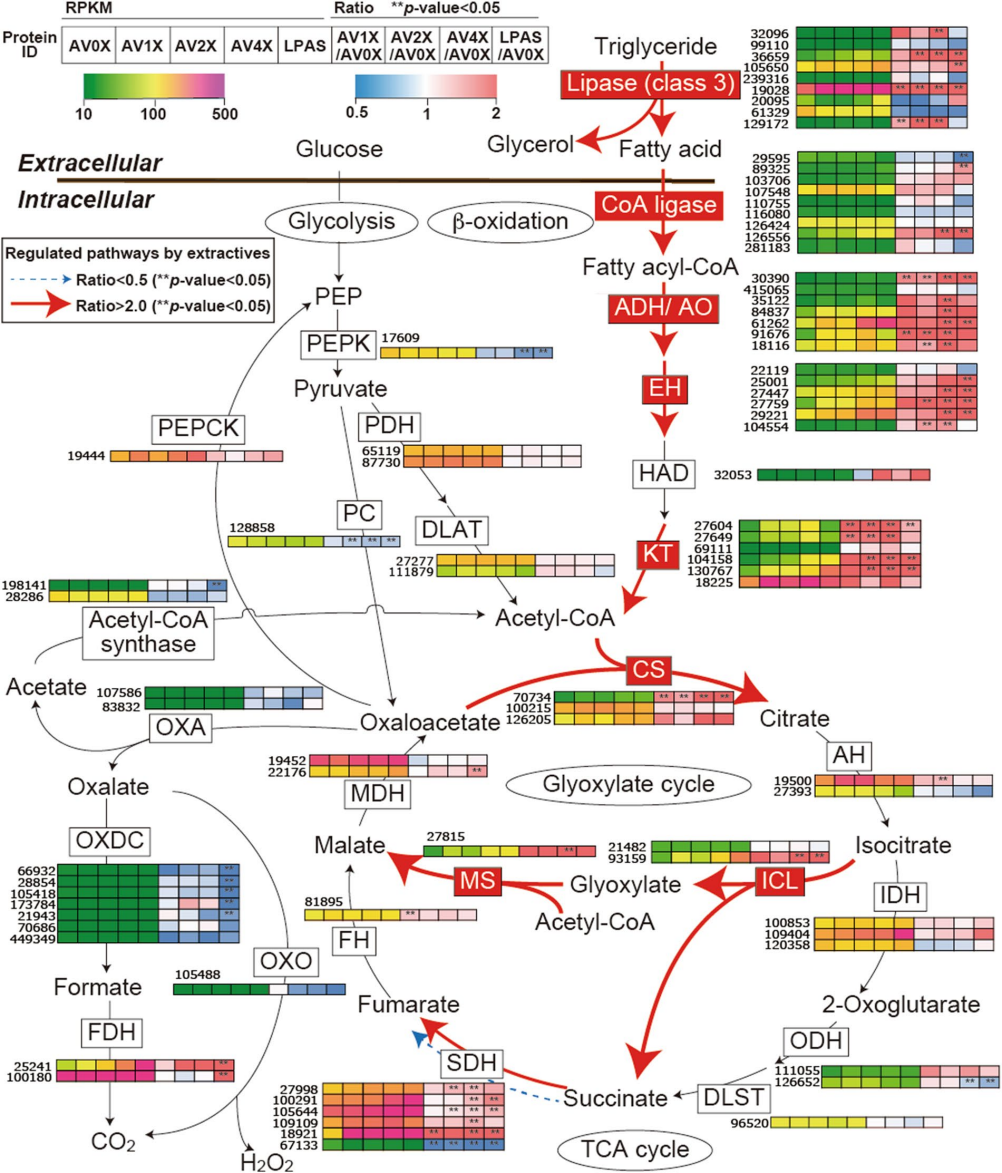
Glyoxylate shunt and proposed relationship to lipid metabolism when *P. gigantea* is cultivated on extracts-containing media (AV1X, AV2X, AV4X) relative to Avicel microcrystalline cellulose medium (AV0X). Enzymes encoded by upregulated genes are red highlighted and associated with thick arrows. Heatmap includes transcriptional expression amount (RPKM) in AV0X, AV1X, AV2X, AV4X and LPAS media and ratio with respect to AV0X. *ADH/AO* acyl-CoA dehydrogenase/oxidase, *AH* aconitate hydratase, *CoA ligase* long fatty acid-CoA ligase, *DLAT* dihydrolipoyllysine-residue acetyltransferase, *DLST* dihydrolipoyllysine-residue succinyltransferase, *EH* enoyl-CoA hydratase, *FDH* formate dehydrogenase, *FH* fumarate hydratase, *GO* glyoxylate oxidase, *KT* ketothiolase (acetyl-CoA C-acyltransferase), *HAD* 3-hydroxyacyl-CoA dehydrogenase, *ICL* isocitrate lyase, *IDH* isocitrate dehydrogenase, *MDH* malate dehydrogenase, *MS* malate synthase, *ODH* oxoglutarate dehydrogenase, *OXA* oxaloacetase, *OXDC* oxalate decarboxylase, *OXO* oxalate oxidase, *PC* pyruvate carboxylase, *PDH* pyruvate dehydrogenase, *PEP* phosphoenolpyruvate, *PEPCK* phosphoenolpyruvate carboxykinase, *PEPK* phosphoenolpyruvate kinase, *SDH* succinate dehydrogenase.

An integrated pathway of lipid metabolism was proposed in a previous study^13^. Based on our current transcript analysis, we mapped the genes on each reaction in the pathway (Fig. 3). The genes involved in lipid metabolism were mostly up-regulated in the presence of extractives including AV1X, AV2X, AV4X and LPAS compared to microcrystalline cellulose (AV0X) as a sole carbon source. However, there were some difference in expression patterns between extractive-coated microcrystalline cellulose and pine wood substrate such as lipase Phlgi_129172 and CoA ligase Phlgi_2959. Among nine lipases, four lipase-encoding transcripts were significantly up-regulated (Fig. 3). Of these, lipase Phlgi_19028 transcripts were most abundant (RPKM = 1767 in AV4X) and accumulated 4.64-fold over AV0X. These results strongly suggest that the gene encoding lipase, which contains a signal peptide, was induced for the extracellular lipid degradation. Further, genes involved in β-oxidation, TCA cycle, and GLOX cycle were activated by the presence of extractives as previously observed in pine wood powder^13^. Thus, the fatty acid existing in the extractives or generated from diglycerides, and/or triglycerides were immediately metabolized through β-oxidation together with the glyoxylate shunt in the cells. Conversely, genes encoding oxaloacetase (OXA), which converts oxaloacetate in the TCA cycle into oxalate, were not up-regulated in the presence of extractives. This observation was different from the comparative transcriptome results of pine wood powder relative to glucose^13^. Considered together with the limited CAZy gene regulation, use of extractive-coated microcrystalline cellulose seems well suited for investigating genetic regulation of degradative processes without the complexities inherent in woody substrates.

### Activities and sequences of *P. gigantea* enzymes in culture filtrates

**Figure 4:**
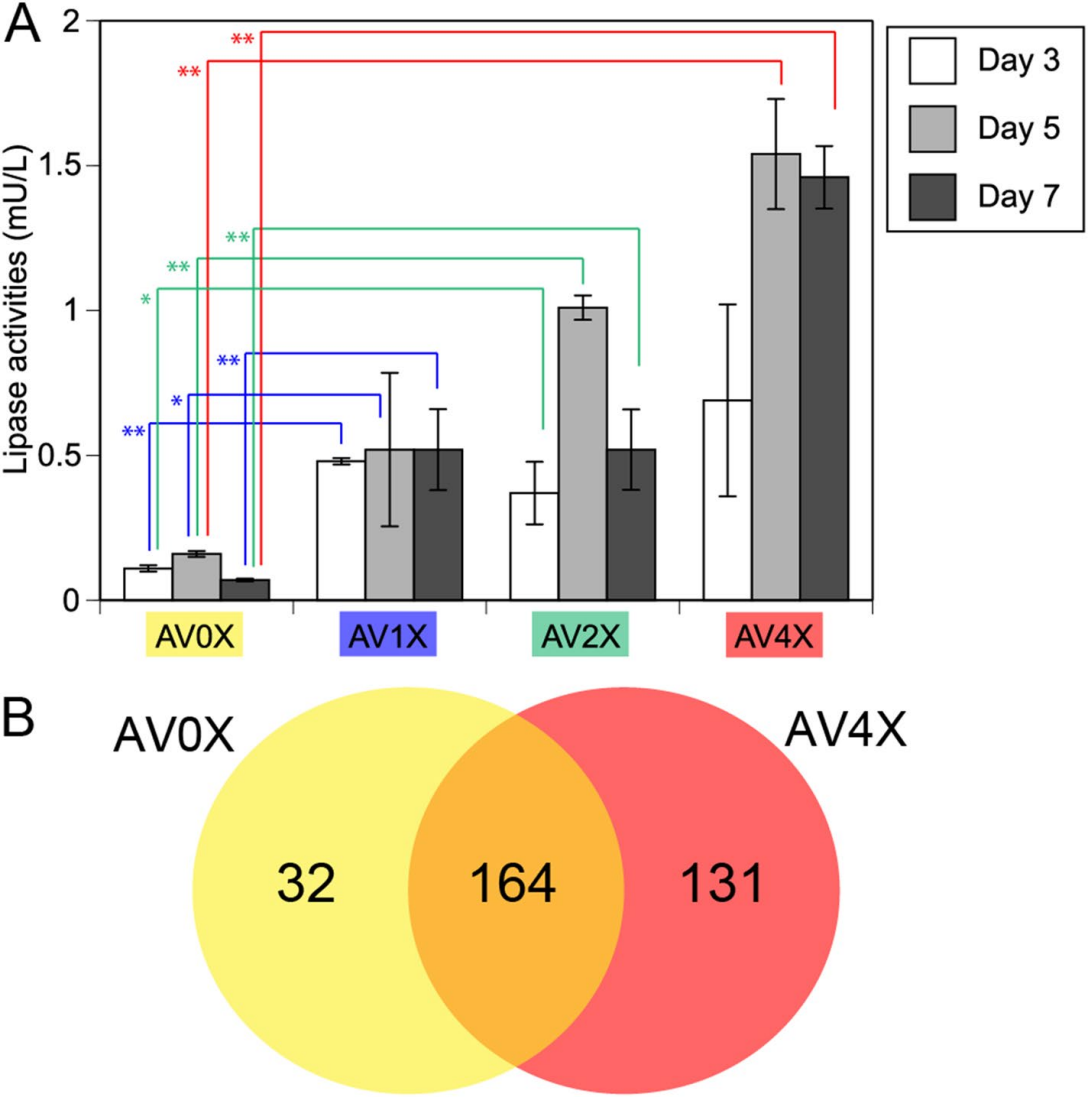
Time course of lipase activities in the secretomes of *Phlebiopsis gigantea* grown on AV0X, AV1X, AV2X and AV4X (**A**). Student’s t-tests were performed; **P value < 0.05, *P value < 0.1. The venn diagram of secretomes of *Phlebiopsis gigantea* grown on AV0X and AV4X based on LC–MS/MS identifications (**B**).

To monitor lipid degradation by *P. gigantea* in response to increasing amounts of extractives, the time course of lipase activity was measured in culture supernatants from AV0X, AV1X, AV2X, and AV4X, using *p*-nitrophenol-dodecanoate (pNPD) as the C12 substrate which is one of model compounds of lipase activity. Significantly increased lipase activities were observed in the culture supernatant of AV1X, AV2X, and AV4X media during time course, compared to AV0X (Fig. 4A). To identify the specific enzyme(s) responsible for this lipase activity, AV0X and AV4X culture filtrates together with AV1X and AV2X were analyzed by LC–MS/MS (Fig. 4B; Supplemental data file S1). A total of 196 and 295 proteins were identified in the AV0X and AV4X proteomes, respectively. Identified proteins met our criteria that a minimum of two unique peptides per protein be identified. Exponentially modified protein abundances (emPAI)^19^ were calculated (summarized in Supplemental data file S1). Among a total of 327 proteins, 32 and 131 proteins were identified only in AV0X and AV4X, respectively, and most of AV4X specific proteins were intracellular proteins including TCA cycle and fatty acid metabolism probably due to the stress of wood extractives. Among 164 common proteins in AV0X and AV4X, 35 were substantially upregulated in the presence of extractives (> threefold). They included Phlgi_19028 lipase (emPAI value = 0.9 and 3.0 in AV0X and AV4X, respectively) together with other secreted enzymes such as Phlgi_28251 GH28 polygalacturonase (emPAI value = 25 and 138 in AV0X and AV4X respectively). The result indicated that Phlgi_19028 lipase likely plays an important role in the degradation of extracellular lipid.

### Structure, phylogenetic analyses and heterologous expression of lipase Phlgi_19028

**Figure 5:**
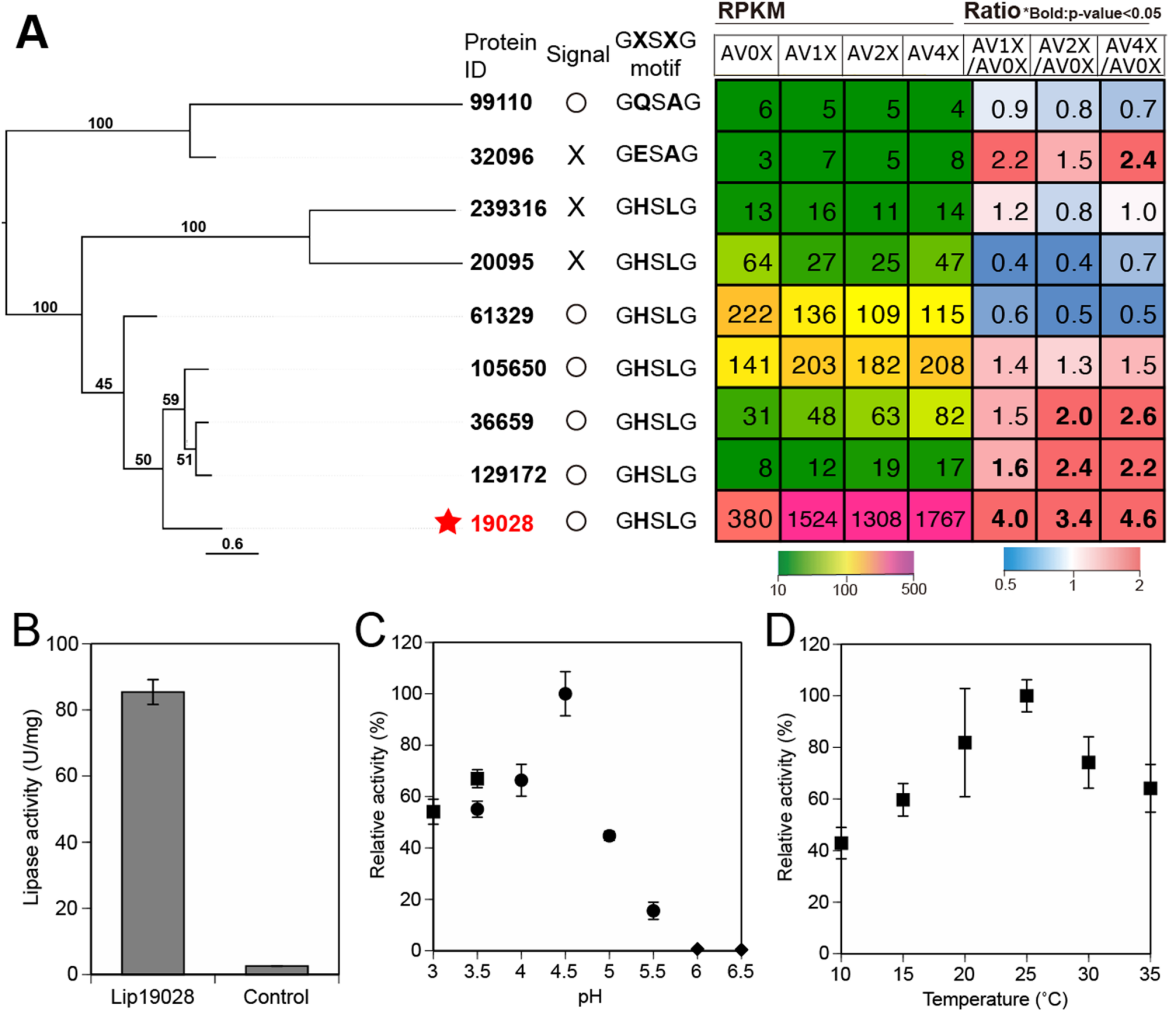
Phylogenetic tree of amino acids of nine lipases encoded in *P. gigantea* genome with gene expression (**A**). ClustalW alignments and RaxML with bootstrap was used for tree construction. Signal, “○” indicates secretion signal peptide at N-terminal, while “x” indicates lack of secretion signal; *secreted protein detected by LC–MS/MS; G-X-S-X-G motif conserved in lipases. (GQSAG and GESAG, found in other Basidiomycetes such as PleoLip369, PleoLip241^22^ and lip2^50^ from *Pleurotus.* Note: Protein model Phlgi_99110 contains an extended intervening sequence. Lipase activities of recombinant PgLip19028 and vector control (**B**). Lipase activities of were measured using pNPD as a substrate in acetate buffer (pH 5.0) for 10-min incubation at 26.5 °C. Optimal pH (**C**) and temperature (**D**) activities of recombinant PgLip19028. pNPD was used as a substrate. Reaction temperature for optimal pH was 25 °C, and reaction pH for optimal temperature was acetate buffer (pH 4.5). Square, tartrate buffer; circle, acetate buffer; diamond, phosphate buffer. Activities of vector control were subtracted.

To further characterize Phlgi_19028, the recombinant lipase was produced in a *Pichia pastoris* expression system. Previous reports demonstrated that *E. coli* expressed lipases localized in inclusion bodies^20^, but *Pichia* produced functional lipases in the soluble fractions^21,22^. Of nine *P. gigantea* lipases, Phlgi_32096 and Phlgi_99110 were the most distantly related but featured the conserved motif, GQSAG and GESAG, found in basidiomycetes lipases^22^ (Fig. 5A). Clustal analyses showed other lipases formed separate, well-defined clades with the GHSLG motif^23–25^. The predicted amino acid sequences of the highly expressed Phlgi_19028 featured the conserved pentapeptide and catalytic triads (Ser180, Asp237, and His251) (Fig. S4). A secretion signal, three N-glycosylation, and one O-glycosylation sites were also predicted.

**Figure 6:**
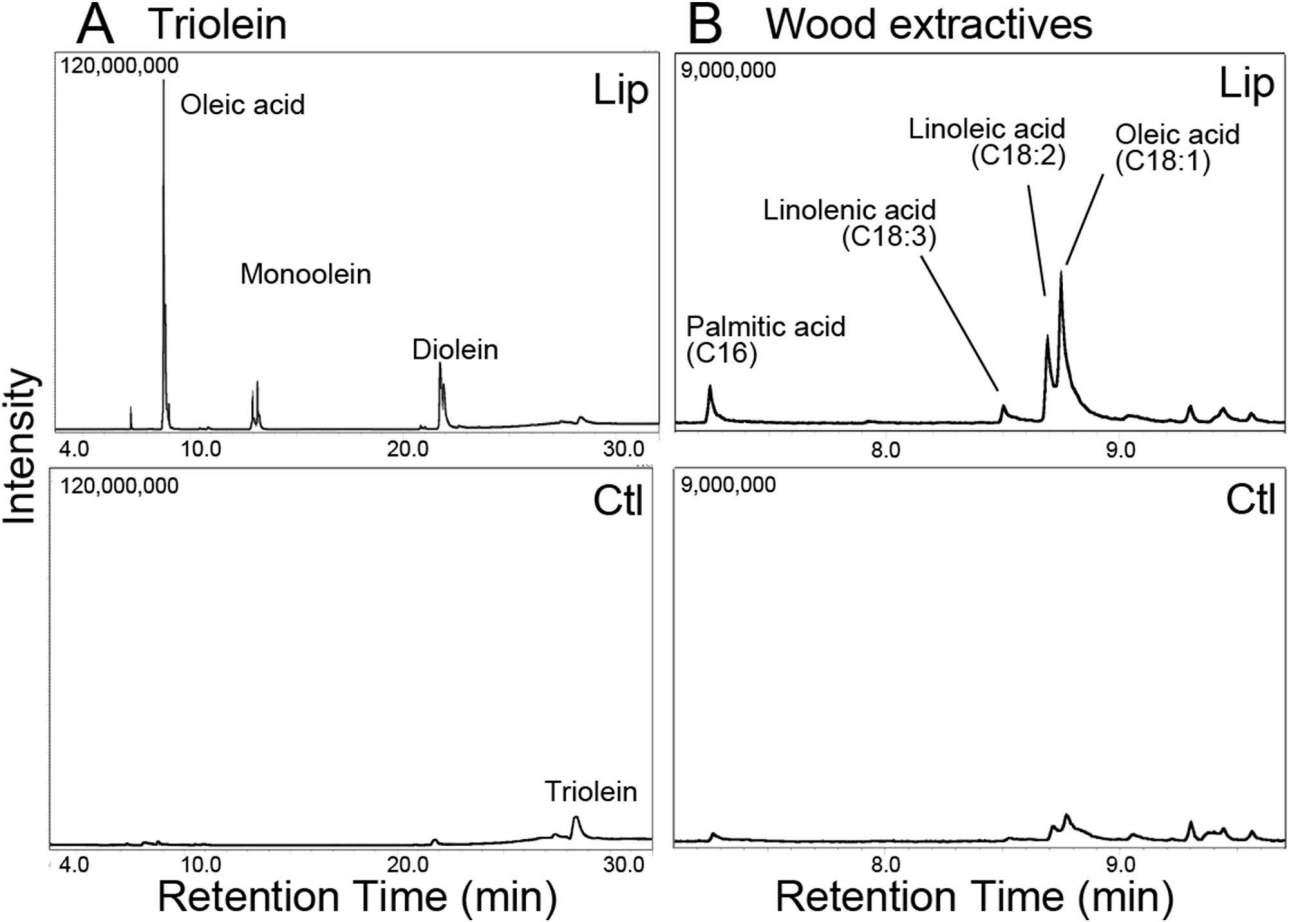
GC–MS analysis of the released products from triolein (**A**) and wood extractives (**B**) after 17 h-incubation with the recombinant lipase Phlgi_19028 (PgLip19028) or vector control (Ctl) expressed by *P. pastoris* in acetate buffer (pH4.5) and at 25 °C.

The recombinant lipase Phlgi_19028 was successfully produced as a major protein in *P. pastoris* culture supernatants (Fig. S5, lane 1) relative to the pPICZα vector control (lane 3). After deglycosylation, the lipase matched the calculated molecular weight (minus glycosylation and signal peptide) of approximately 30.0 kDa as estimated by SDS-PAGE (lane 2). Indeed, significantly higher lipase activity (85.41 ± 3.75 U/mg) toward model compound pNPD was detected compared to a vector control (2.55 ± 0.06 U/mg) (Fig. 5B). We herein named this enzyme PgLip19028. Because some lipases have been reported to increase their activities in the presence of metal ions^26^, we examined the effect of metal ions on recombinant PgLip19028. One mM of nine different metals was added to the assays, and no increases were observed (Table S3). Using the pNPD substrate, the optimal reaction temperature and pH were determined to be 25 °C and pH 4.5, respectively (Fig. 5C,D). Notably, the optimal pH 4.5 is apparently lower than other lipases^22,26^. PgLip19028 was reacted with triolein under the optimized conditions, and the reaction end products were analyzed by TLC and GC–MS analyses. As a result, a peak area of triolein appeared to decrease after the reaction, while peaks of diolein, monoolein, and oleic acid were identified as the end products (Fig. 6A; Fig. S6A). Under the conditions tested, 2.2 mg (≒7.8 µmol) of oleic acid was released from 10 mg (≒11.3 µmol) of triolein after 17 h of reaction. In addition, PgLip19028 released oleic acid, linoleic acid, linolenic acid, and palmitic acid from triglycerides and diglycerides in the coniferous extractives (Fig. 6B; Fig. S6B). In contrast, 0.10 mg (≒0.35 µmol) of oleic acid and 0.16 mg (≒0.58 µmol) of linoleic acid were released from approximately 1 mg of triglycerides included in 10 mg extractives after 17 h of reaction. These indicated that lipase activity toward extractives was lower than that toward triolein probably because concentration of triglycerides was low and other substances of the extractives inhibited the activity**.** Additional measurements over time under various conditions may clarify the precise mechanism. Overall, these results showed that this lipase produces a broad range of fatty acids with different carbon chain lengths and unsaturated bonds distinct from lipids and other lipophilic compounds in coniferous extractives, and that it could play a central role in the degradation of extractives.

## Discussion

The white rot basidiomycete *P. gigantea* used in this study was isolated from *P. taeda* (loblolly pine), which is one of the most common conifer species and heavily used as lumber and pulpwood. *P. gigantea* is known to spread fast and deeply on softwood stumps preventing growth of softwood pathogens. Previous work on *P. gigantea* revealed a distinctive repertoire of genes involved in a unique and efficient system for degrading all components of softwood. Transcriptome analyses had identified hundreds of potential CAZymes, many of which were upregulated on *P. taeda* wood relative to media containing glucose as the sole carbon source^13^. Extending transcript analyses to pine devoid of acetone soluble extractives, comparative analyses suggested that unknown extractives in *P. taeda* influenced the regulation of a diverse array of *P gigantea* genes^13^. In this study, *P. gigantea* was shown to utilize microcrystalline cellulose as a sole carbon source and induced various CAZymes like other white-rot fungi^37,38^. To more precisely identify the genes involved in the response to the extractives, we examined the *P. gigantea* transcriptome and proteome when grown on defined microcrystalline cellulose media supplemented differing amounts of *P. taeda* sapwood extractives. Transcripts encoding protein Phlgi_19028 accumulated sevenfold in non-extracted wood (LPAS) relative to microcrystalline cellulose (AV0X) in this study (Supplemental data file S2). Also, pathways involved in the intracellular metabolism of fatty acids, such as fatty acid β-oxidation, the TCA cycle, and the glyoxylate shunt were up-regulated (Fig. 3). Furthermore, extracellular lipase activities were induced by the extractives (Fig. 4A). These results suggest that lipase activity plays a key role in the metabolism of softwood extractives and that PgLip19028 is responsible for the lipase activity. On the other hand, OXA, which interacts with the TCA cycle and the glyoxylate shunt, was up-regulated by softwood materials in the previous study^13^, but was not induced in this study with microcrystalline cellulose and acetone softwood extractives (Fig. 3). This suggests that OXA associated with oxalic acid accumulation may be induced by unidentified wood components such as cellulose, hemicellulose, lignin, and their degradation products, but not by extractives. In addition to extracellular and intracellular lipid metabolism, CAZymes involved in the degradation of pectin and other polysaccharides (members of GH families 11, 12, 28) were significantly up-regulated by the presence of extractives. This suggested that removal of pectin and other polysaccharides by these enzymes is a first step for efficient deconstruction of extractives-coated wood materials.

*P. gigantea*’s gene expression patterns reveal multiple strategies of this fungus for overcoming the challenging composition of resin-coated microcrystalline cellulose, i.e. ABC transporter (Phlgi1_130987) possibly involved in tolerance to terpenes, glutathione S-transferase (Phlgi_101998), aldehyde dehydrogenase (Phlgi_121047), and aryl-alcohol dehydrogenase (Phigi_89048) in the transformation and detoxification of extractives (Supplemental data file S2). Notably, the significantly upregulated P450s may oxidize aliphatic and/or aromatic chemicals such as those occurring in plant defense systems and thus may be involved in the transformation and degradation of wood extractives. Cytochrome P450 transcripts, especially Phlgi_20511, Phlgi_20514, Phlgi_82811, and Phlgi_68457, were substantially induced by pine extractives (Fig. 2C; Supplemental data file S2). These upregulated transcripts belonged to the reported P450 families CYP52 (CYP5150A), CYP53 (CYP53C), CYP67 (CYP5035A), and CYP503 (CYP512B), respectively^13^. CYP52 transcripts which were the most abundant among the extractive-induced P450s are known to oxidize alkyl chains such as in *n*-alkanes, fatty acids, and alkyl-substituted aromatics^27,28^. In biochemical studies, members of CYP5150A and CYP5150B, upregulated in this study, are known to hydroxylate n-alkyl-substituted benzoic acid and a number of plant defense chemicals including resin acids (e.g. dehydroabietic acid), flavonoids (e.g. flavone) and coumarins (e.g. 7-ethoxycoumarin)^29^. Likewise, induced P450 genes CYP512B (CYP503 family), and CYP5035A (CYP67 family) were shown to catalyze oxidation of these plant defense chemicals. CYP53 family represented is known to hydroxylate benzoate and its derivatives^30^. Beyond these simpler aromatic compounds, P450 monooxygenases CYP512, CYP5141, and CYP5150 have also been shown to oxidize more complex aromatic compounds including polyaromatics and heteroaromatics^31^. Considering their regulation and catalytic potential, *P. gigantea* P450s likely play a role in the metabolism of *P. taeda* extractives.

Based on the sequence annotation, Phlgi1_19028 was predicted to encode lipase in the triacylglycerol hydrolases (E.C. 3.1.1.3) (Fig. 5A). The recombinant PgLip19028 showed a different molecular weight, optimal pH and temperature activities, and substrates specificities than the previously characterized lipases from basidiomycetes^20–22,26,32,33^. Especially, the optimal pH 4.5 is apparently lower than other lipases, but it seems consistent with *P*. *gigantea’s* external environment, which is usually acidic due to secreted organic acids such as oxalic acid^34^. Additionally, from our biochemical characterization, this novel lipase releases various unsaturated fatty acids from *Pinus* extractives at ambient temperature under acidic conditions (Fig. 6). Hence, PgLip19028 is thought to efficiently produce unsaturated fatty acids from lipophilic extractives, allowing *P. gigantea* to proficiently degrade softwood.

Lipases, α/β hydrolases (abH), are classified into 38 abH subfamilies based on amino acid sequences and 3D structures (http://www.led.uni-stuttgart.de/). PgLip19028 is categorized in abH23 subfamily, which includes lipases reported from various pathogens, i.e. *Aspergillus flavus and Fusarium graminearum,* and some of these lipases are involved in penetration to the host organisms^23,24^. In conifers, extractives occur in resin canals and the surrounding parenchyma cells in sapwood, which structurally prevent fungal developments within cell lumens^35^. In addition, it has been reported that larger amounts of triglycerides were extracted from sapwood, compared to heartwood in *Pinus* species^9^. The growth rate of *P. gigantea* greatly varies depending on softwood species, i.e., it grows well on *Pinus* species but not on spruce, larch and fir^36^. Therefore, PgLip19028 is thought to play important roles in the penetration into *Pinus* sapwood by *P. gigantea*, and there would be value in examining *P. gigantea* responses to different extractives from various softwood species in future studies. Considering the wide sequence conservation (Table S4), orthologs of this lipase may generally function in the degradation of lipophilic extractives by wood decay fungi. Further genetic studies, comparative expression, and biochemical studies of this lipase within wood decay fungi will be needed for elucidating more physiological roles in the interaction between wood and fungi.

## Methods

### Culture conditions

The *P. gigantea* isolate 11061-1 was collected from loblolly pine (*P. taeda*) in Macon County, Alabama and sequenced (https://mycocosm.jgi.doe.gov/Phlgi1/Phlgi1.home.html) as described^13^. For each treatment, a single-basidiospore derivative of 11061-1 strain 5–6 was cultivated in triplicate on 250 mL basal salts medium^37,38^ containing: (i.) 1.25 g ground (1 mm mesh) debarked loblolly pine sapwood that had been suspended in 10 mL acetone and subsequently dried in a roto-evaporator (LPAS); or (ii.) 1.25 g microcrystalline cellulose (Avicel PH101, 50uM, Fluka Chemika, Switzerland) that had been suspended in 10 mL acetone and subsequently dried in a roto-evaporator (AV0X); or (iii.) 1.25 g microcrystalline cellulose that had been suspended in 10 mL of an acetone soxhlet extract of 1.25 g, 2.5 g or 5 g loblolly pine and subsequently dried to reconstitute the extractive content at the same (AV1X), two times (AV2X) and four times (AV4X) the native loblolly pine extract concentration, respectively. All loblolly pine was ground from the same 22 cm cross sectional bolt containing 16 annual rings at waist height. After debarking, the material was primarily composed of sapwood. The use of plant parts in the present study complies with international, national and/or institutional guidelines. The composition of basal salts medium contained, per liter, 2 g of NH_4_NO_3_, 2 g of KH_2_PO_4_, 0.5 g of MgSO_4_·7H_2_O, 0.1 g of CaCl_2_·2H_2_O, 1 mg of thiamine hydrochloride, and 10 mL of mineral solution. Mineral solution contained, per liter, 1.5 g of nitrilotriacetic acid, 3 g of MgSO_4_·7H_2_O, 0.5 g of MnSO_4_·H_2_O, 1 g of NaCl, 0.1 g of FeSO_4_·H_2_O, 0.1 g of CoSO_4_, 0.1 g of CaCl_2_, 0.1 g of ZnSO_4_·7H_2_O, 0.01 g of CuSO_4_, 0.01 g of AlK(SO_4_)2·12H_2_O, 0.01 g of H_3_BO_3_, and 0.01 g of NaMoO_4_·2H_2_O^39^. Cultures with solid materials sinking to the bottom were inoculated with mycelial plugs and incubated at 22 °C in a rotary shaker (150 rpm) for 5 days. The culture filtrate and mycelia were separated after the cultivation, and total RNA were purified from the mycelia by RNeasy Mini Kit (Qiagen) with DNase treatment (Qiagen) as previously described^40,41^. For GCMS-based metabolome analyses, the harvested entire cultures including the substrates and fungal mycelium from all the treatments were frozen in liquid nitrogen and lyophilized.

### Time course of lipase activities in the culture filtrates

*P. gigantea* isolate 11061-1 strain CR5-6 was cultivated in triplicates in the culture medium of AV0X, AV1X, AV2X and AV4X, the culture supernatants were collected after 3, 5 and 7 days of cultivation, and protein concentration in the culture supernatants was measured by Protein Assay (Bio-Rad). To determine lipase activities in the secretomes, *p*-nitrophenol-dodecanoate (pNPD, Sigma-Aldrich) was used as substrate. Two microliter of 75 mM pNPD in dimethylsulfoxide (DMSO) was mixed with 50 µL of culture supernatant and 25 µL of 100 mM acetic acid buffer (pH 5.0) in a total volume of 100 µL. After the incubation at 37 °C for 30 min, 25µL of 100 mM Na_2_CO_3_ was added to stop the reaction. The released pNP amount was measured at 405 nm using the standard curve for pNP (Sigma-Aldrich). One unit of lipase activity was defined as the amount of enzyme releasing one micromole of pNP per a minute.

### RNA analysis methods

The transcriptomes were analyzed using Illumina RNA-Seq. Plate-based RNA sample prep was performed on the PerkinElmer Sciclone NGS robotic liquid handling system using Illumina's TruSeq Stranded mRNA HT sample prep kit utilizing poly-A selected mRNA as outlined by Illumina (https://support.illumina.com/sequencing/sequencing_kits/truseq-stranded-mrna.html). Total RNA starting material was 100 ng per sample and 10 cycles of PCR was used for library amplification. Total RNA qualities were examined by using Fragment Analyzer (Agilent Technologies), and RNA quality numbers (RQN) scores were 7.2 ± 0.3, 7.2 ± 0.8, 8.0 ± 1.7, 6.4 ± 0.2, 6.9 ± 2.7 for AV0X, AV1X, AV2X, AV4X and LPAS, respectively. The prepared libraries were quantified using KAPA Biosystem’s next-generation sequencing library qPCR kit and run on a Roche LightCycler 480 real-time PCR instrument. The libraries were then multiplexed with other libraries and sequencing was performed on the Illumina NovaSeq sequencer using NovaSeq XP V1 reagent kits, S4 flow cell, following a 2x150 indexed run recipe. Sequence data for the libraries were deposited in SRA and assigned accession numbers SRP245000 through SRP245010 and 245022 through 245025.

Illumina reads were filtered and trimmed using for artifacts, RNA spike-in reads, PhiX reads and reads containing any Ns. Quality trimming was performed using the phred trimming method set at Q6. Finally, following trimming, reads under the length threshold were removed (minimum length 25 bases or 1/3 of the original read length—whichever is longer). Filtered reads from each library were aligned to the reference genome using HISAT2 version 2.1.0^42^. Strand-specific coverage was determined using deepTools v3.1^43^ and gene counts were generated using feature Counts^44^. Raw gene counts were used to evaluate the level of correlation between biological replicates using Pearson's correlation and determine which replicates would be used in the differential gene expression analysis. DNAStar module ArrayStar ver.16 (Madison, Wisconsin) was used to visualize the normalized reads and compute differential expression in pairwise comparisons. Unless otherwise indicated, parameters used to call genes differentially expressed between culture conditions were *P* value < 0.02 or *P* value < 0.05 when the stringency is relaxed, with > twofold change and RPKM values > 10. Differential expression of all genes were illustrated in volcano plots in Fig. S3.

### Proteome analysis

With minor modification, NanoLC-MS/MS analysis identified extracellular proteins in culture filtrates as described^45–47^. Filtrates from microcrystalline cellulose cultures, with (AV1X, AV2X, AV4X) or without (AV0X) addition of loblolly pine acetone extract, were filtered after 5 days and analyzed. Filtered proteins were precipitated with 10% (wt/vol) trichloroacetic acid and washed three times in cold acetone before air drying. Total proteins from the pellets were further purified via methanol/chloroform/water partitioning, where chloroform and methanol were added to pellets first, followed by water and allowed to partition with a protein interphase formed between polar and non-polar fraction. After multiple methanol washes, these purified protein preps where ultimately resolubilized in 8 M urea/50 mM NH_4_HCO_3_ (pH8.5)/1 mM TrisHCl.

For NanoLC-MS/MS protein identification of samples, equal amounts of total protein per sample were trypsin/LysC digested, OMIX C18 SPE purified (Agilent Technologies), and finally 2 µg loaded for nanoLC-MS/MS analysis using an Agilent 1100 nanoflow system (Agilent Technologies) connected to a hybrid linear ion trap-orbitrap mass spectrometer (LTQ-Orbitrap ELITE, ThermoFisher Scientific) equipped with an EASY-SPRAY electrospray source. Chromatography of peptides prior to mass spectral analysis was accomplished using a capillary emitter column (PEPMAP C18, 3 µM, 100 Å, 150 × 0.075 mm, ThermoFisher Scientific) onto which 2 µL of purified peptides was automatically loaded. The nanoHPLC system delivered solvents A: 0.1% (v/v) formic acid , and B: 99.9% (v/v) acetonitrile, 0.1% (v/v) formic acid at 0.50 µL/min to load the peptides (over a 30 min period) and 0.3 µL/min to elute peptides directly into the nano-electrospray with gradual gradient from 3% (v/v) B to 20% (v/v) B over 154 min and concluded with 12 min fast gradient from 20% (v/v) B to 50% (v/v) B at which time a 5 min flash-out from 50 to 95% (v/v) B took place. As peptides eluted from the HPLC-column/electrospray source, survey MS scans were acquired in the Orbitrap with a resolution of 120,000, followed by MS2 fragmentation of 20 most intense peptides detected in the MS1 scan from 380 to 1800 m/z; redundancy was limited by dynamic exclusion. Raw MS/MS data were converted to mgf file format using MSConvert (ProteoWizard: Open Source Software for Rapid Proteomics Tools Development) for downstream analysis. Resulting mgf files were used to search against forward and decoyed-reversed *P. gigantea* protein database via the JGI portal (https://genome.jgi.doe.gov/portal/Phlgi1/Phlgi1.download.html) with a list of common lab contaminants (available at ftp://ftp.thegpm.org/fasta/cRAP) to establish False Discovery Rate (23,858 total entries) using in-house *Mascot* search engine 2.2.07 [Matrix Science] with variable methionine oxidation, asparagine and glutamine deamidation, plus fixed cysteine carbamidomethylation. Scaffold (version Scaffold_4.7.5, Proteome Software Inc., Portland, OR) was used for spectral based quantification and to validate MS/MS peptide and protein identifications. Peptide identifications were accepted if they could be established at greater than 80.0% probability to achieve an FDR less than 1.0% by the Scaffold Local FDR algorithm. Protein identifications were accepted if they could be established at greater than 99.0% probability to achieve an FDR less than 1.0% and contained at least 2 identified peptides. Protein probabilities were assigned by the Protein Prophet algorithm^48^. Each detected protein abundance was calculated as exponentially modified protein abundances (emPAI)^19^. Proteins that contained similar peptides and could not be differentiated based on MS/MS analysis alone were grouped to satisfy the principles of parsimony. Sequence data was deposited in PRIDE (PRoteomics IDEntifications Database) and assigned identifier PXD021332 (https://www.ebi.ac.uk/pride/archive/projects/PXD021332).

### GC–MS analysis

About 0.4 g of the lyophilized culture samples (n = 1) of microcrystalline cellulose coated with acetone extracts of loblolly pine were Soxhlet-extracted with acetone for 6 h, dried and redissolved in chloroform for GC–MS analyses. GC–MS analyses were performed with a Varian 3800 chromatograph coupled to an ion-trap detector (Varian 4000) using a medium-length fused-silica DB-5HT capillary column (12 m × 0.25 mm internal diameter, 0.1 µm film thickness) from J&W Scientific, enabling simultaneous elution of the different lipid classes^4^. The temperature program started at 100 °C (1 min), raised to 380 °C at 10 °C min^−1^, and held for 5 min. The transfer line was kept at 300 °C, the injector was programmed from 120 °C (0.1 min) to 380 °C at 200 °C min^−1^, and helium was used as carrier gas at a rate of 2 mL min^−1^. Compounds were identified by mass fragmentography, and by comparing their mass spectra with those of the Wiley and NIST libraries and standards. Quantification was obtained from total-ion peak area, using response factors of the same or similar compounds (palmitic acid, abietic acid, sitosterol, 1,3-dipalmitin, cholesteryl linoleate and tripalmitin). *N*, *O*-Bis(trimethylsilyl)trifuluoroacetamide (BSTFA) in the presence of pyridine was used to prepare trimethylsilyl derivatives.

### Synthesis of the recombinant *P. gigantea* lipase Lip19028

The DNA encoding the class 3 lipase (PgLip19028), minus the native secretion signal, was synthesized by JGI service (Fig. S4). The codon-optimized gene was subcloned into an expression vector of pPICZα (Invitrogen) by using primers for PIPE cloning^49^, PgiLip19028F_PIPE_Pic, TCTCTCGAGAAAAGACTCCCATCGCCTGTCCAC, PgiLip19028R_PIPE_Pic, AGTTTTTGTTCTAGATTAAGTACAATAAATAGTTCCG, PicVector_PIPE_F, TCTAGAACAAAAACTCATCTCAGAAGAGGATCTGAATAGCG, PicVector_PIPE_R, TCTTTTCTCGAGAGATACCCCTTCTTCTTTAGCAGCAATGCTG (underlined sequences were overlapped). The recombinant lipase was produced by transformed *Pichia pastoris* overexpressing Lip19028 following the manufacturer’s instructions (Invitrogen). The culture supernatant of *Pichia pastoris* was applied to saturated ammonium sulfate precipitation, and the resulting protein precipitate was dissolved in 100 mM acetate buffer (pH 5.0). Ten micrograms of crude protein were denatured and digested by Endoglycosidase H (Endo H, New England Biolab) for protein deglycosylation, then protein samples were applied to SDS-PAGE analysis (Bio-Rad), and the gel was stained by Coomassie Brilliant Blue (CBB) R-250 (FUJIFILM Wako Pure Chemical Corporation, Osaka Japan).

### Sequence analyses and phylogenetic trees

Sequences were analyzed by web-based tool Expasy (https://www.expasy.org) as follows. Secretion signal peptides were predicted by SignalP ver 5.0 (http://www.cbs.dtu.dk/services/SignalP/). N-glycosylation and O-glycosylation sites were predicted by NetNGlyc ver 1.0 (http://www.cbs.dtu.dk/services/NetNGlyc/) and NetOGlyc ver 4.0 (http://www.cbs.dtu.dk/services/NetOGlyc/), respectively. The theoretical molecular weight was calculated by compute pI/Mw (https://web.expasy.org/compute_pi/). Secondary structure prediction was performed by Phyre 2 (http://www.sbg.bio.ic.ac.uk/~phyre2/html/page.cgi?id=index). The multiple alignments of nine lipase amino acid sequences from *P. gigantea* and other reported lipases were performed by ClustalW, and a phylogenetic tree was executed by RaxML with bootstrap, and it was drawn by Figtree software.

### The biochemical assay of the recombinant PgLip19028 from *P. gigantea*

Prior to lipase activity determinations, the protein concentration of crude proteins was diluted to 10 µg/mL and then 1 µg/mL of crude protein was mixed with 1.5 mM pNPD in *tert*-butyl methyl ether (tBE), 5% DMSO and 25 mM buffer in total volume of 160 µL. The final concentration of tBE was kept 15%, which showed the highest activity compared to 5%, 10%, 15%, 20%, 25%, and 30% tBE. After the reaction mixture was incubated at the appropriate temperature for 10 min, 40 µL of 100 mM Na_2_CO_3_ was added to stop the reaction. One unit was calculated as described above. For optimal pH determination, tartaric buffer ranging from pH 3.0 to pH 3.5, sodium acetate buffer ranging from pH 3.5 to pH 5.5 and potassium phosphate buffer ranging from pH 6.0 to pH 6.5 were assessed. Lipase activity of PgLip19028 was corrected by subtracting activity in the supernatant of a vector-only control culture. Sensitivity of lipase activity to metals was determined with 1 mM of ZnCl_2_, MnCl_2_·4H_2_O, CoCl_2_·6H_2_O, NiCl_2_·6H_2_O, CuCl_2_·2H_2_O, CdCl_2_, LiCl, CaCl_2_·2H_2_O, MgCl_2_·6H_2_O, or ZnCl_2_. For glycerides degradation assay, 1 µg/mL of crude protein was mixed with 10 mg/mL triolein or *Pinus* extractives in tBE, 5% DMSO and 25 mM acetate buffer (pH 4.5) in total volume of 1000 µL. The reaction mixture was incubated at 25 °C for 17 h with shaking at 120 rpm, lyophilized and the residue dissolved in chloroform. The sample was applied to TLC plates^32^ and the remaining sample analyzed by GCMS after trimethylsilylation by BSTFA.

## Supplementary information

Supplementary Information 1.

Supplementary Information 2.

Supplementary Information 3.
