# Supplementary material for: Omics analyses and biochemical study of Phlebiopsis gigantea elucidate its degradation strategy of wood extractives
Source: Sci Rep. 2021 Jun 15;11:12528. doi: 10.1038/s41598-021-91756-5 (PMC8206109; doi:10.1038/s41598-021-91756-5)
Supplement: Supplementary file 3 — Supplementary Information 3. [file 41598_2021_91756_MOESM3_ESM.pdf]

Supplementary information of

Omics analyses and biochemical study of *Phlebiopsis gigantea* elucidate its degradation strategy of wood extractives

Mana Iwata[a], Ana Gutiérrez[b], Gisela Marques[b], Grzegorz Sabat[c], Philip J. Kersten[d], Daniel Cullen[d] Jennifer M. Bhatnagar[e], Jagjit Yadav[f], Anna Lipzen[g], Yuko Yoshinaga[g], Aditi Sharma[g], Catherine Adam[g], Christopher Daum[g], Vivian Ng[g], Igor V. Grigoriev[gh], Chiaki Hori[a*]

Division of Applied Chemistry. Department of Engineering, Hokkaido University, Sapporo, Hokkaido, 060-8628, Japan[a1]; Instituto de Recursos Naturales y Agrobiología de Sevilla (IRNAS), CSIC, Reina Mercedes 10, E-41012 Seville, Spain[b]; University of Wisconsin Genetics Biotechnology Center, Madison, WI 53706, USA[c]; USDA, Forest Products Laboratory, Madison, WI 53726, USA[d]; Department of Biology, Boston University, Boston, MA 02215, USA[e]; University of Cincinnati, Cincinnati, OH 45267, USA[f]; US Department of Energy Joint Genome Institute, Lawrence Berkeley National Laboratory, One Cyclotron Road, Berkeley, CA 94720, USA[g]; Department of Plant and Microbial Biology, University of California Berkeley, Berkeley, CA 94720, USA[h]

[*]*Corresponding author*: Chiaki Hori, Department of Engineering, Hokkaido University, Sapporo, Hokkaido, 060-8628, Japan, chori@eng.hokudai.ac.jp

Supplemental Table S1. The 20 most abundant genes encoding CAZymes involved in lignocellulose degradation expressed in *P. gigantea* grown on AV0X medium using crystalline cellulose as a sole carbon source.

#	Protein ID	Putative function	Avicel and Loblolly pine (RPKM)					Fold change to relative to AV0X			
			AV0X	AV1X	AV2X	AV4X	LPAS	AV1X	AV2X	AV4X	LPAS
1	34136	GH7 Cellobiohydrolase (CBH) /Endoglucanase (EG)	7447	9513	10484	6241	5133	1.3	1.4	0.8	0.7
2	30921	CBM1	2505	3449	4434	3717	1104	1.4	1.8	1.5	0.4
3	84111	GH5 EG	2470	1885	2719	2191	1158	0.8	1.1	0.9	0.5
4	17701	GH6 CBH/EG	2156	2390	3123	2008	638	1.1	1.4	0.9	0.3
5	18264	AA9-CBM1 lytic monooxygenase (LPMO)	1896	2895	2885	1911	341	1.5	1.5	1.0	0.2
6	85016	GH10 Endo-1,4-beta-xylanase (EX)	1825	1513	2502	1908	1766	0.8	1.4	1.0	1.0
7	110296	GH5-7 Endo-1,4-beta-mannanase	1804	2589	2095	2342	676	1.4	1.2	1.3	0.4
8	31010	CBM1	1588	1157	1461	1287	420	0.7	0.9	0.8	0.3
9	227588	AA9 LPMO	1578	1345	629	611	449	0.9	0.4	0.4	0.3
10	86144	GH5 EG	1117	1469	1657	1386	374	1.3	1.5	1.2	0.3
11	18210	GH1Beta-glucosidase (BGL)	1107	821	853	619	511	0.7	0.8	0.6	0.5
12	33120	GH5 EG	967	670	432	421	150	0.7	0.4	0.4	0.2
13	99876	AA8-AA3_1 Cellobiose dehydrogenase (CDH)	874	1339	2052	1503	683	1.5	2.3	1.7	0.8
14	79150	AA9-CBM1 LPMO	824	1209	1324	913	140	1.5	1.6	1.1	0.2
15	113976	GH11 EX	749	1529	1270	1723	175	2.0	1.7	2.3	0.2
16	124737	GH45 EG	736	271	290	294	64	0.4	0.4	0.4	0.1
17	103464	CE16 Carbohydrate esterase	730	855	741	800	270	1.2	1.0	1.1	0.4
18	61167	GH128 b-glucanase	640	809	696	757	710	1.3	1.1	1.2	1.1
19	80168	GH7 CBH/EG	621	248	370	294	45	0.4	0.6	0.5	0.1
20	69505	GH7 fragment CBH/EG	621	248	370	294	45	0.4	0.6	0.5	0.1

Supplemental Table S2. ***P. gigantea*** **regulated CAZy genes encoding enzymes potentially involved in lignocellulose degradation.**

Protein ID	Putative function	Gene expression level in extractive-coated Avicel crystalline cellulose and loblolly pine cultures (RPKM)					*Fold change to relative to AV0X			
		AV0X	AV1X	AV2X	AV4X	LPAS	AV1X	AV2X	AV4X	LPAS
14295	AA6 1,4-BQR	12	23	27	37	11	1.9	2.4	**3.2**	1.0
18201	Peroxidase HTP	123	41	48	50	207	**0.3**	**0.4**	**0.4**	1.7
21055	GH3 β-glucosidase	70	14	13	20	5	0.2	**0.2**	**0.3**	**0.1**
21241	GH11 β -1,4-xylanase	14	59	59	93	61	**4.1**	**4.1**	**6.5**	4.2
26602	GH17- β -1,3-glucanase	7	56	90	98	21	**8.1**	**12.9**	**14.2**	3.1
27927	GH18 chitinase	11	41	21	24	13	**3.7**	**1.9**	**2.2**	1.2
28251	GH28 Polygalacturonase	88	453	1662	1021	1553	**5.1**	**18.9**	**11.6**	**17.6**
31576	CE10 candidate unknown	44	17	18	11	14	0.4	0.4	**0.3**	0.3
33454	GH13 α-glucanase	50	250	134	217	196	**5.0**	2.7	**4.3**	**3.9**
34479	GH12 Endoglucanase	34	132	228	278	367	3.9	6.8	**8.3**	10.9
36341	GH28 Polygalacturonase	5	13	24	28	75	3.0	**5.2**	**6.2**	**16.6**
71032	PL14 glucuronan-lyase	4	7	8	7	4	**1.7**	**2.2**	1.8	1.1
72251	AA9 LPMO	95	23	8	15	3	0.2	**0.1**	**0.2**	**0.0**
77440	GH31 glycosidase	65	48	40	31	16	0.7	0.6	**0.5**	**0.3**
78526	Peroxidase DyP	136	22	17	25	375	**0.2**	**0.1**	**0.2**	2.8
88425	GH55 Exo- β -1,3 glucanase	24	126	16	25	48	**5.3**	0.7	1.1	2.0
88507	GH18 chitinase	16	61	36	61	19	**3.7**	**2.2**	**3.7**	1.1
101126	AA3_2 GMC oxidoreductase	26	52	51	62	30	**2.0**	**1.9**	**2.4**	1.2
105609	PL14 glucuronan-lyase	7	58	17	23	13	**8.9**	2.6	**3.5**	2.0
108516	AA3_3 Methanol oxidase (MOX)	84	27	29	28	164	0.3	0.3	**0.3**	1.9
118444	AA9 LPMO	33	60	518	163	558	1.8	**15.6**	4.9	**16.8**
125261	CBM12 unknown	570	170	122	141	1185	0.3	**0.2**	**0.2**	2.1
126344	GH12 Endoglucanase	13	27	39	47	51	**2.1**	**3.1**	**3.7**	4.0
128606	AA5_1 cro2-like	399	219	217	183	265	0.5	**0.5**	**0.5**	0.7
130353	GH20 β -hexosaminidase	29	79	68	88	45	2.7	2.3	**3.0**	1.5
359855	CBM13 unknown	82	5	3	5	17	0.1	**0.0**	**0.1**	0.2

* Bold: P-value <0.05, Pink >2-fold, Blue <0.5-fold.

Supplemental Table S3. Metal sensitivity of recombinant PgLip19028[1].

	% Relative activity
None	100 ± 4.8
Cd	90.2 ± 4.3
Li	80.5 ± 2.9
Ca	66.4 ± 7.9
Zn	66.4 ± 12.3
Mg	55.4 ± 5.4
Co	50.9 ± 2.3
Ni	50.4 ± 0.6
Mn	42.2 ± 4.6
Cu	32.4 ± 2.8

[1] 1mM

Supplemental Table S4. Similarity of lipases belonging to abH23 subfamily (Mw approximately 30kDa; GHSLG motif) derived from filamentous fungi and wood rot fungi.

Phylum	Clade	Ecology	Origin	Accession #/ Protein ID	Length (aa)	Identity (%)	Name; Reference
Mucoromycota			*Rhizomucor miehei*	CAA00250	363	24	TGL; (1)
Ascomycota	Eurotiales	Pathogen	*Aspergillus flavus*	AAO17921	306	21	LIPA; (2)
	Hypocreales	Pathogen	*Fusarium graminearum*	AAQ23181	351	38	FGL1; (3)
Basidiomycota	Agaricales	white	*Shizophilum commune*	2641616	311	49	
	Boletales	Brown	*Coniophora puteana*	125023	308	67	
		Brown	*Serpula lacrymans*	1074869	273	71	
	Russulales	white	*Heterobasidion annosum*	317659	307	60	
		white	*Stereum hirsutum*	149961	319	60	
	Polyporales	Brown	*Postia planceta*	108803	321	59	
		Brown	*Wolfiporia cocos*	136660	320	58	
		Brown	*Fomitopsis pinicola*	1021548	324	55	
		white	*Ceriporiopsis suvermispora*	778716	315	63	
		white	*Dichomitus squalens*	139624	302	66	
		white	*Trametes versicolor*	75646	309	61	
		white	*Phanerochaete chrysosporium*	2990121	316	68	
		white	*Phanerochaete carnosa*	265032	317	71	
		white	*Phlebia breviospora*	145263	305	68	
		white	*Phlebiopsis gigantea*	19028	309	-	This study

*Basidiomycete lipases were selected by using blastp search against each wood rot fungal genome deposited in JGI website.

References

1. Brady L, Brzozowski AM, Derewenda ZS, Dodson E, Dodson G, Tolley S, Turkenburg JP, Christiansen L, Huge-Jensen B, Norskov L, Thim L, Menge U. 1990. A serine protease triad forms the catalytic centre of a triacylglycerol lipase. Nature 343:767–770.
2. Yu J, Mohawed SM, Bhatnagar D, Cleveland IE. 2003. Substrate-induced lipase gene expression and aflatoxin production in *Aspergillus parasiticus* and *Aspergillus flavus*. J Appl Microbiol 95:1334–1342.
3. Voigt CA, Schäfer W, Salomon S. 2005. A secreted lipase of *Fusarium graminearum* is a virulence factor required for infection of cereals. Plant J 42:364–375.

Supplemental Figure Legends

Supplementary Figure S1. GC-MS chromatograms of lipophilic compounds (as TMS-ethers) identified in acetone extracts from Avicel crystalline cellulose coated with acetone extracts of loblolly pine without (A) and with (B) *P. gigantea* inoculation. Peak identification, 1: palmitic acid, 2: margaric acid, 3: linoleic acid, 4: oleic acid, 5: stearic acid; 6: pimaric acid, 7: isopimaric acid, 8: palustric acid, 9: levopimaric acid; 10: dehydroabietic acid, 11: 7-oxodehydroabietic acid, 12: sitosterol, 13: 1-palmitoyl-2-oleoylglycerol; 14: 1-oleoyl-2-palmitoylglycerol; 15: 1,2-palmitoylolein, 16: 1,3-palmitoylolein, 17: campesteryl ester; 18: sitosteryl ester, 19: 1,2-dioleinoylpalmitin, 20: triolein.

Supplemental Figure S2. Heat map showing expression values of all 11 891 *P. gigantea* genes when cultured in Avicel crystalline cellulose medium containing with no extract (AV0X), the same medium with increasing amounts of loblolly pine extract (AV1X, AV2X, AV4X) or on loblolly pine without Avicel or extract (LPAS). Log_2 (RPKM) values were used for the clustering analysis by Heatplus in Bioconductor (https://www.bioconductor.org).

Supplementary Figure S3.Volcano plots of all 11,891 *P. gigantea* genes based on gene expression of media containing extractive-coated crystalline cellulose, AV1X, AV2X, AV4X or on loblolly pine including extractive (LPAS), relative to crystalline cellulose without extractive (AV0X). Red circle, significantly up-regulated genes (>2-fold, p-value<0.05); Blue circle, significantly down-regulated genes (<1/2-fold, p-value<0.05); Gray circle, not significant genes.

Supplementary Figure S4. Primary and secondary structure of Phlgi_19028 lipase. Underlined sequences at N-terminal, secretion signal peptides; Red-highlighted S or T, N-glycosylation; Red-highlighted N, O-glycosylation. Bold pentapeptides, G-X-S-X-G motif conserved in lipases; Three blue-highlighted amino acids (Ser, Asp, His), catalytic triads. Theoretical pI/Mw of mature enzyme without a signal peptide, 5.00 / 30.0 kDa.

Supplementary Figure S5. SDS-PAGE of recombinant PgLip19028. Lane 1 and 2, loaded with lipase without/with treatments by Endo H, respectively; Lane 3 and 4, Vector controls without/with Endo H, respectively; Lane 5, Endo H only.

Supplementary Figure S6. TLC analysis of the released products from triolein (A) and wood extractives (B) after incubation with lipase Phlgi_19028 or vector control (Ctl) in acetate buffer (pH 4.5) and at 25˚C overnight.

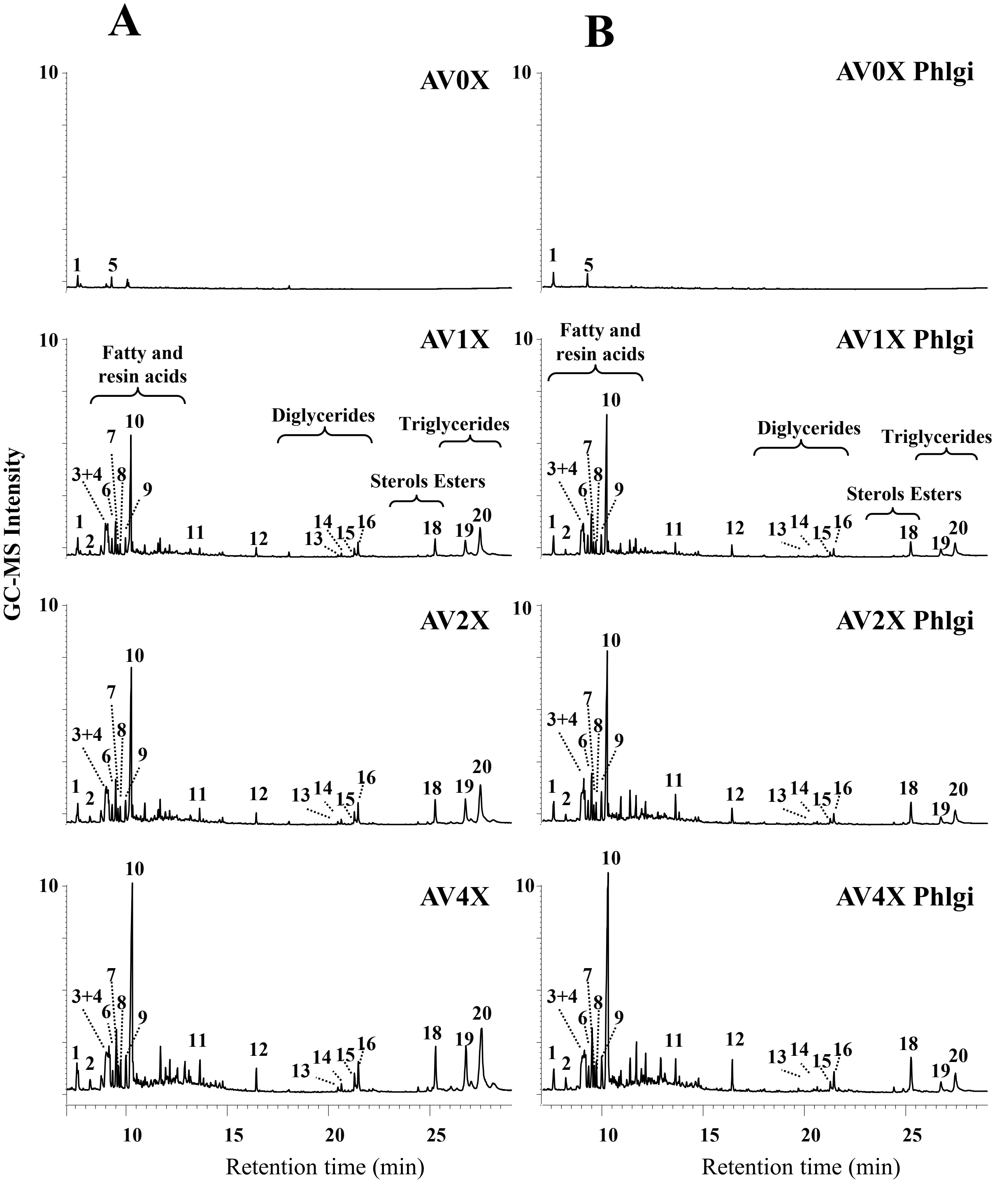

Supplemental Figure 1

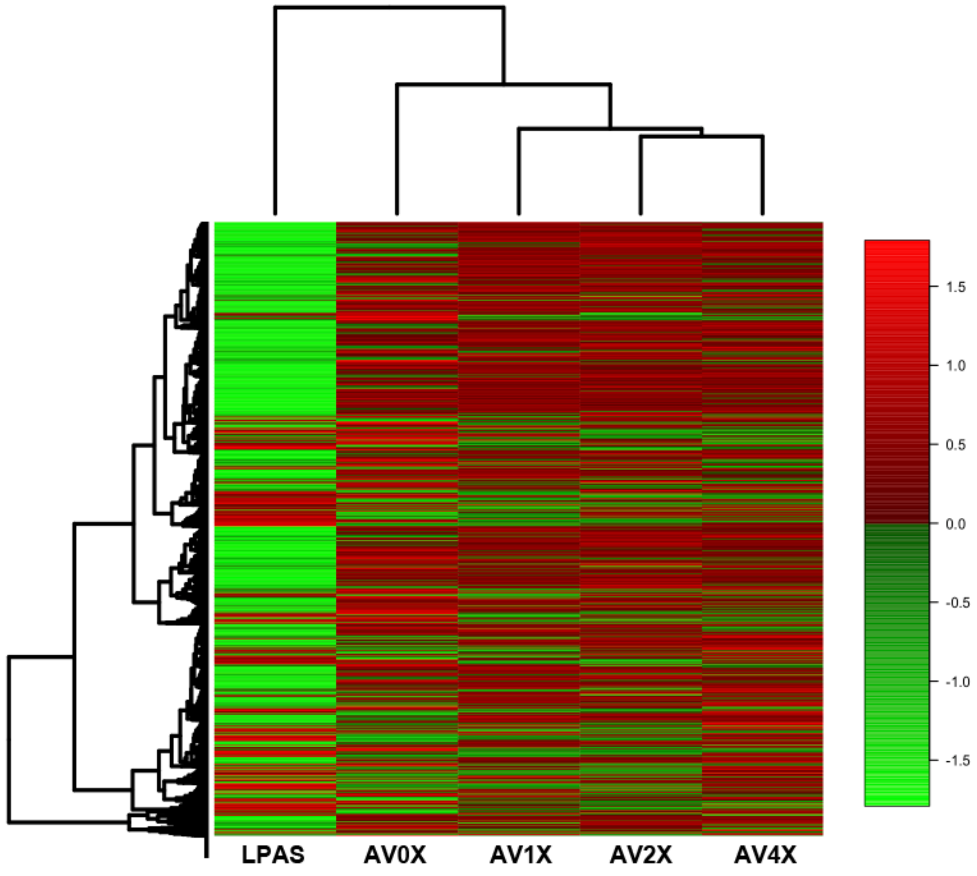

Supplementary Figure S2

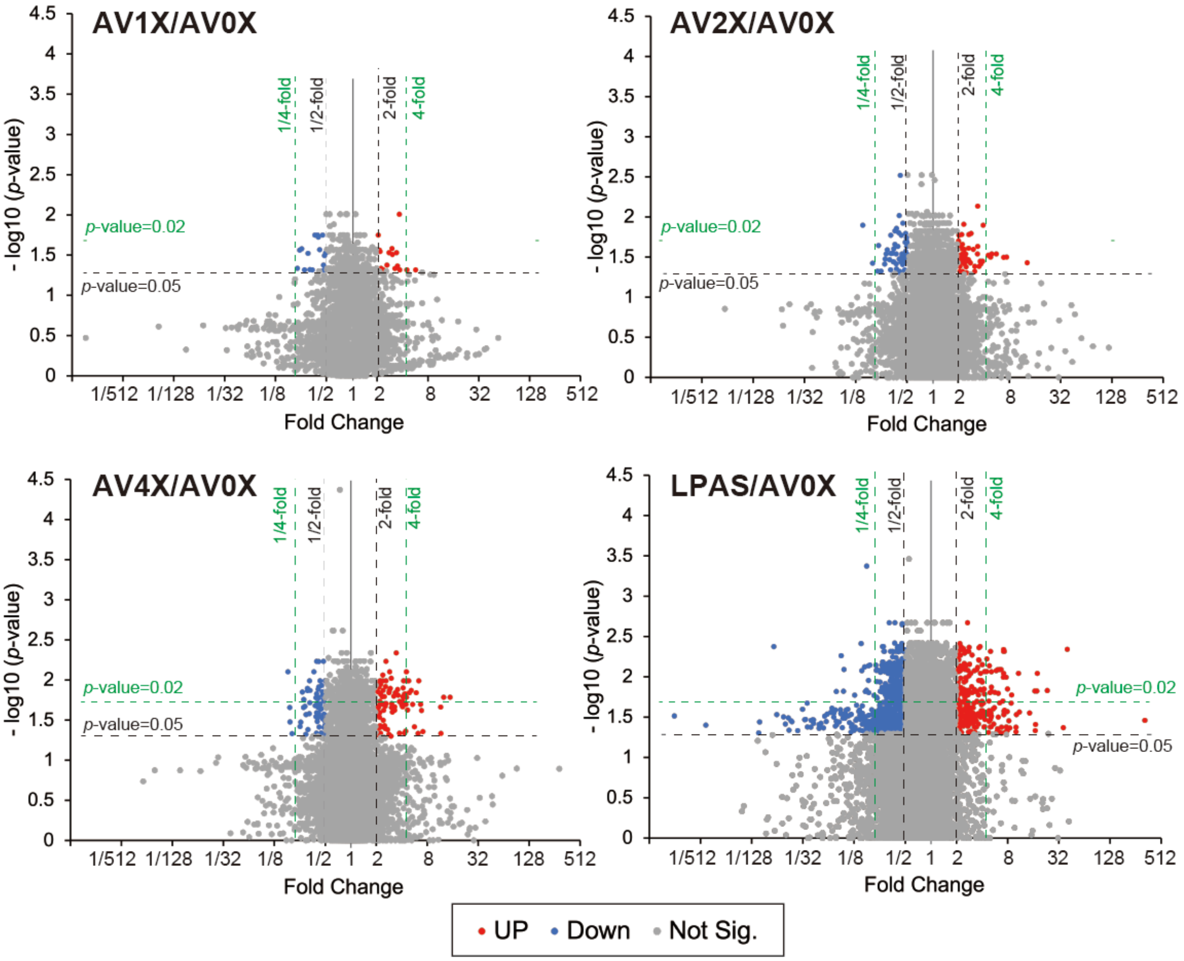

Supplementary Figure S3

1 MISLFSRLAV VAVAAYGVQA LPSPVHPEFP RDGVSTLSAA
41 TLAGLAPFTQ FARAAYCPSS KVTGWKCGQA CAAVPGFEVS
81 LTGGDGNGVQ LFYVGYWPAQ NSVVVAHEGT DPTQLLSDLT
121 DANIAMSGLD ATLFPGVPSS VKGHAGFIAE HAKTAATILA
161 EVKRLISSTG ATQVTLV**GHS** **LG**GALAELES LFMTLNLPSS
201 IHVKGVTYGT PRVGNPAFAT YFDSKVADFE RINNESDPVP
241 IVPGRGLGFS HVHGEI IVA PDDAVSCPGD DDATDAQCTI
281 STVPTLLESN LLNHLGPYQG IYIGTIYCT

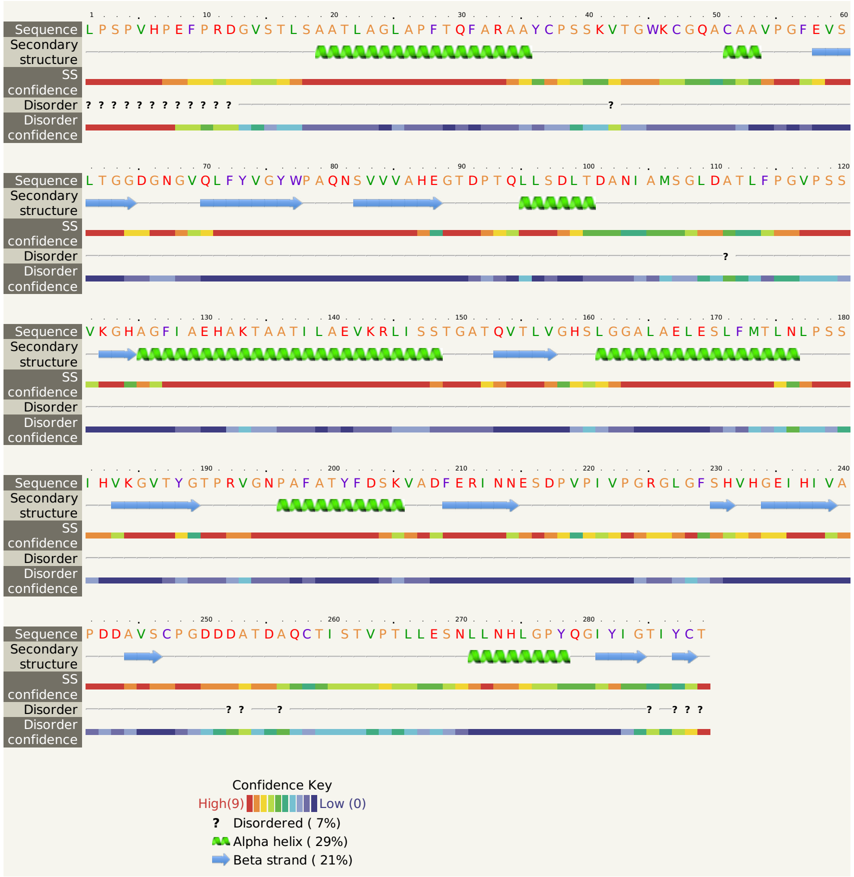

Supplementary Figure S4.

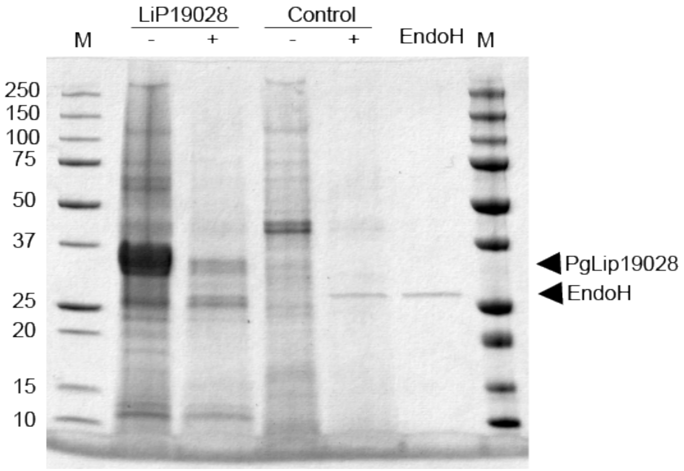

Supplementary Figure 5.

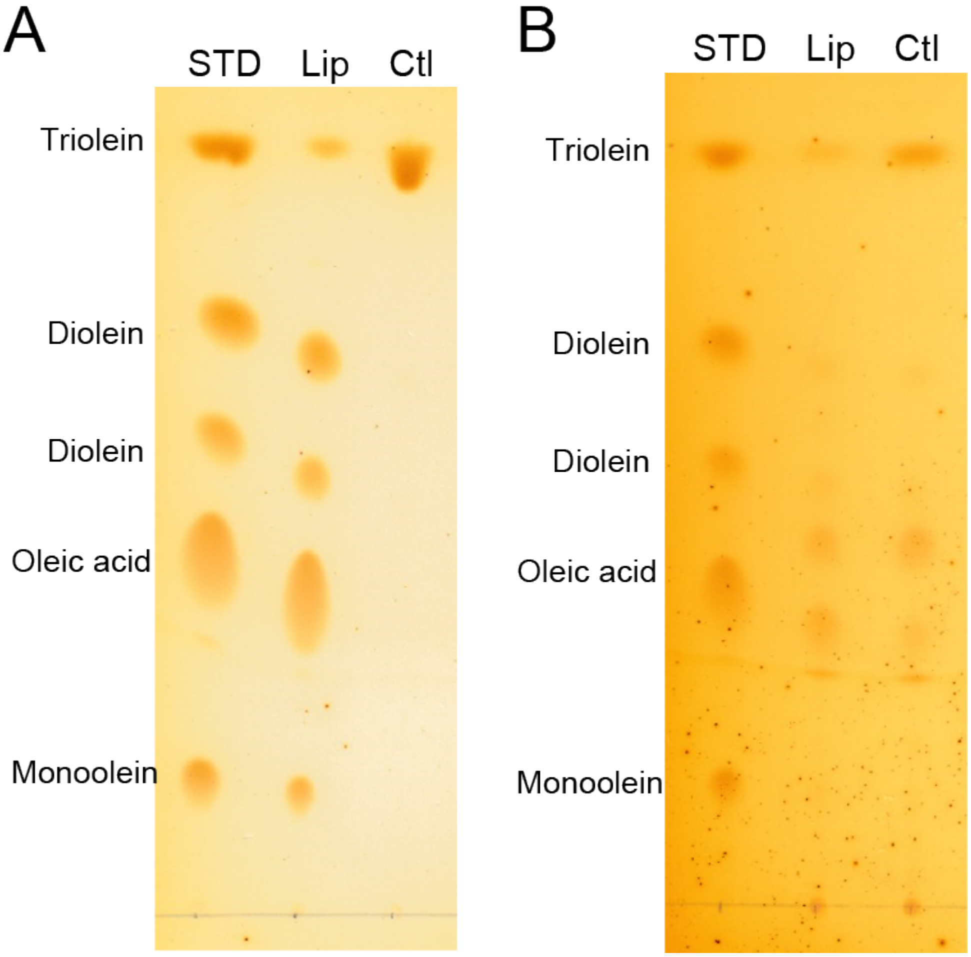

Supplementary Figure S6.
